# Machine Learning–Based Asthma Attack Prediction Models From Routinely Collected Electronic Health Records: Systematic Scoping Review

**DOI:** 10.2196/46717

**Published:** 2023-12-07

**Authors:** Arif Budiarto, Kevin C H Tsang, Andrew M Wilson, Aziz Sheikh, Syed Ahmar Shah

**Affiliations:** 1 Asthma UK Center for Applied Research Usher Institute University of Edinburgh Edinburgh United Kingdom; 2 Bioinformatics and Data Science Research Center Bina Nusantara University Jakarta Indonesia; 3 Norwich Medical School University of East Anglia Norwich United Kingdom; 4 Norfolk and Norwich University Hospital NHS Foundation Trust Norwich United Kingdom

**Keywords:** asthma attack, exacerbation, prognosis, machine learning, electronic health record, review, EHR, asthma

## Abstract

**Background:**

An early warning tool to predict attacks could enhance asthma management and reduce the likelihood of serious consequences. Electronic health records (EHRs) providing access to historical data about patients with asthma coupled with machine learning (ML) provide an opportunity to develop such a tool. Several studies have developed ML-based tools to predict asthma attacks.

**Objective:**

This study aims to critically evaluate ML-based models derived using EHRs for the prediction of asthma attacks.

**Methods:**

We systematically searched PubMed and Scopus (the search period was between January 1, 2012, and January 31, 2023) for papers meeting the following inclusion criteria: (1) used EHR data as the main data source, (2) used asthma attack as the outcome, and (3) compared ML-based prediction models’ performance. We excluded non-English papers and nonresearch papers, such as commentary and systematic review papers. In addition, we also excluded papers that did not provide any details about the respective ML approach and its result, including protocol papers. The selected studies were then summarized across multiple dimensions including data preprocessing methods, ML algorithms, model validation, model explainability, and model implementation.

**Results:**

Overall, 17 papers were included at the end of the selection process. There was considerable heterogeneity in how asthma attacks were defined. Of the 17 studies, 8 (47%) studies used routinely collected data both from primary care and secondary care practices together. Extreme imbalanced data was a notable issue in most studies (13/17, 76%), but only 38% (5/13) of them explicitly dealt with it in their data preprocessing pipeline. The gradient boosting–based method was the best ML method in 59% (10/17) of the studies. Of the 17 studies, 14 (82%) studies used a model explanation method to identify the most important predictors. None of the studies followed the standard reporting guidelines, and none were prospectively validated.

**Conclusions:**

Our review indicates that this research field is still underdeveloped, given the limited body of evidence, heterogeneity of methods, lack of external validation, and suboptimally reported models. We highlighted several technical challenges (class imbalance, external validation, model explanation, and adherence to reporting guidelines to aid reproducibility) that need to be addressed to make progress toward clinical adoption.

## Introduction

### Background

Asthma is a chronic lung illness characterized by reversible airway blockage caused by inflammation and narrowing of the small airways in the lungs that can lead to cough, wheezing, chest tightness, and breathing difficulties [1]. It is a common noncommunicable disease that affects children and adults alike. In 2019, asthma affected an estimated 262 million individuals, resulting in 461,000 fatalities [1,2]. Asthma attacks occur particularly in those with poorly controlled diseases [3]. An asthma attack is a sudden or gradual deterioration of asthma symptoms that can have a major influence on a patient’s quality of life [4]. Such attacks can be life-threatening and necessitate rapid medical attention, such as an accident and emergency department visit or hospitalization, and can even lead to mortality [5]. Asthma attacks are prevalent, with >90,000 annual hospital admissions in the United Kingdom alone [6]. Early warning tools to predict asthma attacks offer the opportunity to provide timely treatments and, thereby, minimize the risk of serious outcomes [4].

Machine learning (ML) offers the potential to develop an early warning tool that takes different risk factors as input and then outputs the probability of an adverse outcome. So far, logistic regression (LR) has been the most common approach in building an asthma attack risk prediction tool [7-9]. However, the predictive performance of this method may be inferior to more advanced ML methods, especially for relatively high-dimensional data with complex and nonlinear relationships between the variables [10,11]. The use of ML has been investigated in a wide range of medical domains by using various data such as electronic health records (EHRs), medical images, genomics data, and wearables data [12-14]. However, to the best of our knowledge, there is still no widely used ML-based asthma attack risk prediction tool in clinical practice.

### Objective

Previous recent systematic reviews have discussed the choice of models used for asthma prognosis [15,16]. An ML pipeline, however, has several components besides modeling choice (eg, feature engineering [17]), which can profoundly influence the performance of the algorithms. Owing to the lack of consensus about what constitutes best practices for the application of ML for predicting asthma attacks, there is considerable heterogeneity in previous studies [15,16], thereby making direct comparisons challenging. In this scoping review, we aimed to critically examine existing studies that used ML algorithms for the prediction of asthma attacks with routinely collected EHR data. Besides data type and choice of models, we have reviewed additional ML pipeline challenges. These include customizing *off-the-shelf* algorithms to account for domain-specific subtleties and the need for the model to be explainable, extensively validated (externally and prospectively), and transparently reported.

## Methods

### Overview

The scoping review was conducted based on the 5-stage framework by Arksey and O’Malley [18]. This framework includes identifying the research question; searching and collecting relevant studies; study filtering; data charting; and finally, collating, summarizing, and reporting the results. The research questions in this scoping review were the following:

1. What methods are commonly used in developing an asthma attack prediction model?
2. How did the authors process the features and outcome variables?
3. Are there any of these prediction models that have been implemented in a real-world clinical setting?

We then translated these 3 questions into the patient or population, intervention, comparison, and outcomes model [19,20], as shown in Table 1.

**Table 1:** The patient or population, intervention, comparison, and outcomes structure.

Item	Expansion	Keywords
P	Patient, population	People with asthma
I	Intervention, prognostic factor, or exposure	Machine learning
C	Comparison of intervention	N/A^a^
O	Outcome	Asthma attack

^a^N/A: not applicable.

### Search Strategy

We used the patient or population, intervention, comparison, and outcomes model in Table 1 as our framework for defining relevant keywords. This approach led us to include clinical terms associated with asthma attacks, encompassing concepts such as asthma exacerbation, asthma control, asthma management, and hospitalization. In addition, we integrated technical terminology related to ML, incorporating terms such as artificial intelligence, supervised methods, and deep learning (DL). All the keywords that we used in the search strategy can be found in Multimedia Appendix 1 [4,11,21-35]. Overall, 2 databases, PubMed and Scopus, were chosen as the sources of papers. The search period was between January 1, 2012, and January 31, 2023, and the search was limited to the title, abstract, and keywords of each paper but without any language restriction. The complete query syntax for both databases is listed in Textbox 1.

**Query syntax.**

**Scopus**

- ((TITLE-ABS-KEY(“asthma”) AND (TITLE-ABS-KEY(“management”) OR TITLE-ABS-KEY(“control”) OR TITLE-ABS-KEY(“attack”) OR TITLE-ABS-KEY(“exacerbation”) OR TITLE-ABS-KEY(“risk stratification”) OR TITLE-ABS-KEY(“risk prediction”) OR TITLE-ABS-KEY(“risk classification”) OR TITLE-ABS-KEY (hospitalization”) OR TITLE-ABS-KEY (“hospitalisation”) OR TITLE-ABS-KEY (“prognosis”))) AND (TITLE-ABS-KEY(“machine learning”) OR TITLE-ABS-KEY(“artificial intelligence”) OR TITLE-ABS-KEY(“supervised method”) OR TITLE-ABS-KEY(“unsupervised method”) OR TITLE-ABS-KEY (“deep learning”) OR TITLE-ABS-KEY (“supervised learning”) OR TITLE-ABS-KEY (“unsupervised learning”))) AND PUBYEAR > 2011

**PubMed**

- ((asthma[Text Word]) AND ((Management[Text Word]) OR (Control[Text Word]) OR (Attack[Text Word]) OR (Exacerbation[Text Word]) OR (Risk Stratification[Text Word]) OR (Risk Prediction[Text Word]) OR (Risk Classification[Text Word]) OR (hospitalization[Text Word]) OR (hospitalisation[Text Word]) OR (prognosis[Text Word])) AND ((machine learning[Text Word]) OR (Artificial Intelligence[Text Word]) OR (supervised method[Text Word]) OR (unsupervised method[Text Word]) OR (deep learning[Text Word]) OR (supervised learning[Text Word]) OR (unsupervised learning[Text Word]))) AND (“2012/01/01”[Date - Publication] : “2023/01/31”[Date - Publication])

### Eligibility Criteria and Study Selection

Overall, 2 authors (AB and KCHT) performed the 2-step study selection process. During the first selection step, we focused on the abstract. In the second step, we conducted a thorough reading of the full text of the manuscript. We only included papers that met our inclusion criteria: (1) used asthma attack as the outcome, (2) included an ML-based prediction model, and (3) used EHR data as the main data source. We defined the concept of EHR-derived data as structured, text-based, individual-level, and routinely collected data gathered within the health care system. In cases of unclear information extracted from the abstract, the reviewers decided to retain the studies for the next iteration (full-text review). We excluded nonresearch papers, such as commentary and systematic review papers because of the insufficient technical information. We also filtered out papers that did not provide sufficient details about the ML approach and the result, including protocol papers.

### Data Extraction

From each of the eligible papers, we extracted data from the full text and web-based supplements. We then summarized these data under different categories such as data set (whether publicly available or not), population characteristics (source, size, age range, and region), year of data, outcome definition and how it was represented in the model, number of features, feature selection method, imbalance handling strategy, ML prediction methods, performance evaluation metric, evaluation result, external validation, explainability method, and real-world clinical setting implementation. The data extraction and summarization for each paper were conducted independently by 2 authors (AB and KCHT). In case of any discrepancies, the 2 authors discussed them in detail during face-to-face meetings to reach an agreement. If the 2 reviewers could not resolve the disagreement, we had a further discussion with the whole team. For each study, we have reported both the performance evaluation result of the prediction models and the most important predictors where available.

## Results

### Overview

**Figure 1:**
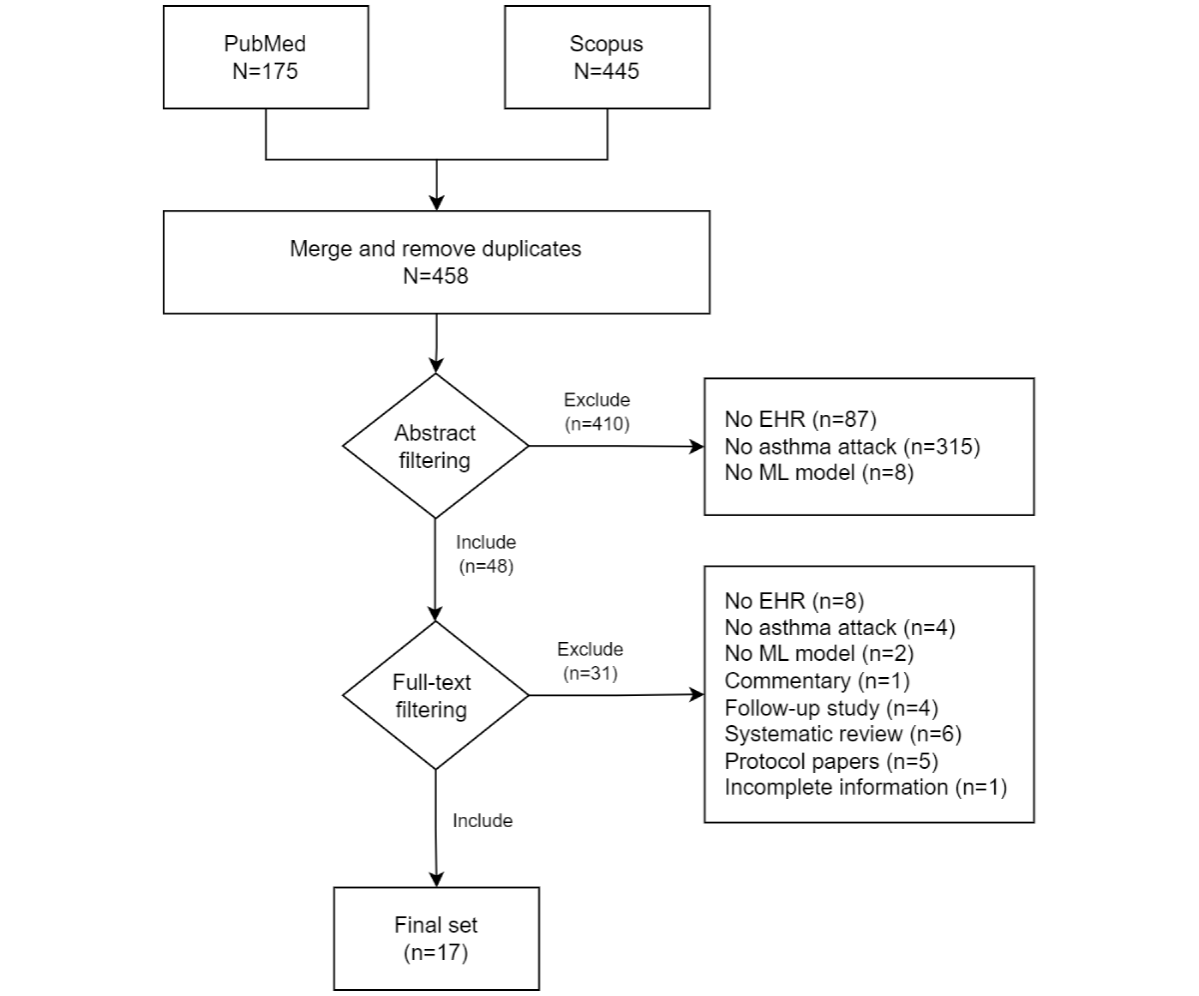
PRISMA (Preferred Reporting Items for Systematic Reviews and Meta-Analyses) diagram. EHR: electronic health record; ML: machine learning.

In total, 458 nonduplicated, potentially eligible papers were identified. At the end of the selection process, 3.7% (17/458) of the papers were included based on the inclusion criteria (refer to the PRISMA [Preferred Reporting Items for Systematic Reviews and Meta-Analyses] diagram in Figure 1). The earliest study that was included in the full review was published in 2018. In the abstract filtering stage, most of the studies (353/458, 77.1%) were excluded because the prediction outcome was not an asthma attack. We included 10.5% (48/458) of the studies in the full-text filtering stage. Eventually, 3.1% (14/458) of the studies were excluded because they did not meet our inclusion criteria. Then, 2.6% (12/458) nonresearch papers were also excluded. In addition, we excluded 0.9% (4/458) of the studies, which were a follow-up for 2 main papers that we included in the extraction stage. All the summary points in these follow-up studies were identical to the ones in the main studies. We also excluded 0.2% (1/458) of the studies owing to insufficient information.

### Asthma Data Sets

Table 2 summarizes the basic information about each included study. Only 6% (1/17) of the studies used routinely collected data from primary care alone [21]. Of the 17 studies, only 8 (47%) used data from secondary care, and the remaining 8 (47%) used routinely collected data from both primary and secondary care. All studies used data sets hosted either at the author’s institution or their collaborators’ institution, except a study [22] that used publicly available data (the Medical Information Mart for Intensive Care III data set [36]) as one of their data sets. Overall, 76% (13/17) of the studies used only EHR data to build the prediction model. Of the 17 studies, 4 (24%) studies integrated EHR data with additional modalities, including radiology images (chest computed tomography scans) [23] and environmental data [11,24,25], aiming to enhance predictive accuracy. The study populations varied across the studies, with most of them involving adults (8/17, 47%), followed by the general population, both children and adults (5/17, 29%), and children (4/17, 24%). Of the 17 studies, 14 (82%) had study populations from the United States. The other countries studied included Japan, Sweden, and the United Kingdom. All studies incorporated >1000 samples, except a study [23] that trained the prediction model on <200 samples. Among the studies, the biggest data set had data from 397,858 patients [26].

**Table 2:** Summary of studies’ basic information.

Study, year	Health care setting	Publicly available data set	Data source	Region	Data year	Sample size
Inselman et al [27], 2022	Secondary care	No	Single modality	United States	2003-2020	3057
Hurst et al [25], 2022	Both	No	Multimodality	United States	2014-2019	5982
Hogan et al [28], 2022	Secondary care	No	Single modality	United States	2013	18,489
Zein et al [29], 2021	Both	No	Single modality	United States	2010-2018	60,302
Sills et al [30], 2021	Secondary care	No	Single modality	United States	2009-2013	9069
Hozawa et al [31], 2021	Secondary care	No	Single modality	Japan	2016-2017	42,685
Lisspers et al [32], 2021	Both	No	Single modality	Sweden	2000-2013	29,396
Ananth et al [23], 2021	Secondary care	No	Multimodality	United Kingdom	2018-2020	200
Tong et al [33], 2021	Both	No	Single modality	United States	2011-2018	82,888
Mehrish et al [24], 2021	Secondary care	No	Multimodality	United States	2013-2017	10,000
Xiang et al [4], 2020	Both	No	Single modality	United States	1992-2015	31,433
Cobian et al [34], 2020	Both	No	Single modality	United States	2007-2011	28,101
Luo et al [35], 2020	Both	No	Single modality	United States	2005-2018	315,308
Roe et al [22], 2020	Secondary care	Yes	Single modality	United States	2001-2012	38,597
Luo et al [26], 2020	Both	No	Single modality	United States	2012-2018	397,858
Wu et al [21], 2018	Primary care	No	Single modality	United States	1997-2002	4013
Patel et al [11], 2018	Secondary care	No	Multimodality	United States	2012-2015	29,392

### Data Preprocessing

There was considerable heterogeneity in the definition of the prediction outcome used in the models, including asthma exacerbation [4,25,27,29,31,32,34], asthma-related hospitalization [11,24,26,30,33,35], asthma readmission [28], asthma prevalence [24], asthma-related mortality [22], and asthma relapse [21].

The time horizon used to define the prediction outcome also varied across studies. Of the 17 studies, 6 (35%) studies defined the model task as a 1-year prediction [4,23,26,31,33,35]. Other variations in the time horizon for the outcome were 180 days [28], 90 days [34], 28 days [29], and 15 days [32]. A study compared the prediction model performances across 3 time horizons: 30, 90, and 180 days [25]. Of the 17 studies, 2 (12%) studies undertook a different approach, where the aim was to predict asthma attack–related hospitalization within 2 hours after an accident and emergency department visit [11,30]. Of the 17 studies, 3 (18%) studies did not report the prediction time horizon [21,22,24].

There was an obvious class imbalance in 76% (13/17) of the studies (Table 3). Class imbalance is a problem where the distribution of samples across the classes is skewed [37]. Ignoring this problem during model development will produce a biased model. Among the selected studies, the smallest minority class ratio accounted for as little as 0.04% [32]. Among these 17 studies, only 5 (29%) [4,21,30,32,33] explicitly mentioned their strategies to appropriately handle imbalanced data. Synthetic minority oversampling technique [38], oversampling [39,40], and undersampling [39,40] were the methods reported in these studies. The objective of these 3 methods is to balance the proportion of samples in each class by either generating synthetic data from the minority class or omitting a certain number of samples in the majority class. Of the 17 studies, only 2 (12%) studies used a balanced data set [22,23], whereas 2 (12%) other studies did not report the class ratio of their data set [24,34]. Various feature selection methods were explicitly mentioned as part of the data preprocessing step, including backward stepwise variable selection [28], light gradient boosting method feature importance [32], and Pearson correlation [32]. Of the 17 studies, 5 (29%) studies [4,26,30,33,35] implemented the feature selection process as the built-in method in the model development phase, whereas the remaining studies did not mention the feature selection method in their report. The smallest feature set used in the study was 7 variables [24], and the biggest set was >500 variables [32]. The handling of missing values varied across the studies. In most cases (9/17, 53%), missing values were treated either as a distinct category or assigned a specific value [21,23,25-27,29,32,33,35]. However, some studies opted to exclude data containing missing values [4,11,28,30], whereas others did not specify their approach for addressing this issue [22,24,31,34]. Notably, more than half of the studies (11/17, 65%) did not describe their methods for data normalization. This step is particularly critical for certain ML algorithms such as LR and support vector machine to prevent uneven weighting of features in the model. In contrast, 35% (6/17) of the studies [11,22,23,26,33,35] used a standard mean normalization technique to standardize the continuous features, ensuring uniform scaling across the data set.

**Table 3:** Summary of the data preprocessing step.

Study, year	Outcomes	Prediction time horizon	Class imbalance ratio (%)	Data imbalance handling methods	Feature selection methods	Number of features
Inselman et al [27], 2022	Asthma exacerbation	180 d	22.60	Unknown	Unknown	21
Hurst et al [25], 2022	Asthma exacerbation	30, 90, and 180 d	37	Unknown	Unknown	41
Hogan et al [28], 2022	Asthma readmission	180 d	5.70	Unknown	Backward stepwise variable selection	21
Zein et al [29], 2021	Asthma exacerbation	28 d	Nonsevere=32.80 Severe=2.90	Unknown	Unknown	82
Sills et al [30], 2021	Asthma-related hospitalization	Admission after A&E^a^ department visit	22.50	Oversampling	Automated feature selection	13
Hozawa et al [31], 2021	Asthma exacerbation	365 d	13.70	Unknown	Unknown	25
Lisspers et al [32], 2021	Asthma exacerbation	15 d	0.04	Undersampling and weighting method	Correlation and LGBM^b^ model	>500
Ananth et al [23], 2021	Asthma exacerbation	365 d	50	Unknown	Unknown	17
Tong et al [33], 2021	Asthma-related hospitalization or A&E department visit	365 d	1.66	WEKA^c^	Automated feature selection	234
Mehrish et al [24], 2021	Asthma prevalence, asthma-related hospitalization, or hospital readmission	Unknown	Unknown	Unknown	Unknown	7
Xiang et al [4], 2020	Asthma exacerbation	365 d	7.20	SMOTE^d^	Automated feature selection	Unknown
Cobian et al [34], 2020	Asthma exacerbation	90 d	Unknown	Unknown	Unknown	>25
Luo et al [35], 2020	Asthma-related hospitalization	365 d	3.59	Unknown	Automated feature selection	235
Roe et al [22], 2020	Asthma-related mortality	Unknown	49	Unknown	Unknown	42
Luo et al [26], 2020	Asthma-related hospitalization	365 d	2.30	Unknown	Automated feature selection	337
Wu et al [21], 2018	Asthma relapse	Unknown	32.89	Random undersampling	Unknown	60
Patel et al [11], 2018	Asthma-related hospitalization	Admission after ED^e^ visit	17	Unknown	Unknown	100

^a^A&E: accident and emergency.

^b^LGBM: light gradient boosting method.

^c^WEKA: Waikato Environment for Knowledge Analysis.

^d^SMOTE: synthetic minority oversampling technique.

^e^ED: emergency department.

### ML Methods and Performance Evaluation

Table 4 describes the ML and performance evaluation methods used in the selected studies. We found a wide range of ML methods in the selected studies. Most (14/17, 82%) used conventional ML methods such as support vector machine [41], random forest [42], naïve Bayes [43], decision tree (DT) [44], K-nearest neighbor [45], and artificial neural network [46]. LR and its variations (ie, Ridge, Lasso, and Elastic Net) [47] were found to be the most common baseline model among the studies (10/15, 67%) [4,11,22-25,27-30,32,34]. Some studies developed the prediction model with more advanced ML algorithms such as gradient boosting DT (GBDT)–based methods [11,22,25-27,29,31-33,35] and DL-based methods [4,21,34]. A few studies [26,30,35] also used automated model selection tools, such as Waikato Environment for Knowledge Analysis [48] and autoML [49]. GBDT-based methods including extreme gradient boosting (XGBoost) [50] were the common best-performing models (area under the curve scores ranging from 0.6 to 0.9). The model performances in all studies were evaluated using the area under the receiver operating characteristic curve score, except in a study [21] that used *F*_1_-score as the only performance metric. Half of them (9/17, 53%) included additional evaluation metrics such as accuracy, precision, recall, sensitivity, specificity, positive predictive value, negative predictive value, *F*_1_-score, area under the precision-recall curve, and microaveraged accuracy [21,23,25-27,30,32,33,35]. Owing to different data sets and the heterogeneity in the definitions of the outcome, prediction time horizon, and preprocessing across the studies, we considered a direct comparison across studies based on the reported evaluation metric to be inappropriate. Only 18% (3/17) of the studies included external validation using retrospective studies in their analysis pipeline [21,26,33].

**Table 4:** Summary of machine learning (ML) methods.

Study, year	ML methods	Best models	Best performance metrics	External validation
Inselman et al [27], 2022	GLMNet^a^, RF^b^, and GBM^c^	GBM	AUC^d^=0.74	No
Hurst et al [25], 2022	Lasso, RF, and XGBoost^e^	XGBoost	30-d AUC=0.761 90-d AUC=0.752 180-d AUC=0.739	No
Hogan et al [28], 2022	Cox proportional hazard, LR^f^, and ANN^g^	ANN	AUC=0.636	No
Zein et al [29], 2021	LR, RF, and GBDT^h^	GBDT	Nonsevere AUC=0.71 Hospitalization AUC=0.85 ED^i^ AUC=0.88	No
Sills et al [30], 2021	AutoML, RF, and LR	AutoML	AUC=0.914	No
Hozawa et al [31], 2021	XGBoost	XGBoost	AUC=0.656	No
Lisspers et al [32], 2021	XGBoost, LGBM^j^, RNN^k^, and LR (Lasso, Ridge, and Elastic Net)	XGBoost	AUC=0.90	No
Ananth et al [23], 2021	LR, DT^l^, and ANN	LR	AUC=0.802	No
Tong et al [33], 2021	WEKA^m^ and XGBoost	XGBoost	AUC=0.902	Yes
Mehrish et al [24], 2021	GLM^n^, correlation models, and LR	LR	AUC=0.78	No
Xiang et al [4], 2020	LR, MLP^o^, and LSTM^p^ with an attention mechanism	LSTM with an attention mechanism	AUC=0.7003	No
Cobian et al [34], 2020	LR, RF, and LSTM	LR with L1 (Ridge)	AUC=0.7697	No
Luo et al [35], 2020	WEKA and XGBoost	XGBoost	AUC=0.859	No
Roe et al [22], 2020	XGBoost, NN^q^, LR, and KNN^r^	XGBoost	AUC=0.75	No
Luo et al [26], 2020	WEKA and XGBoost	XGBoost	AUC=0.820	Yes
Wu et al [21], 2018	LSTM	LSTM	Binary classification F1-score=0.8508 Multiclass classification F1-score=0.4976	Yes
Patel et al [11], 2018	DT, Lasso, RF, and GBDT	GBDT	AUC=0.84	No

^a^GLMNet: Lasso and Elastic-Net Regularized Generalized Linear Models.

^b^RF: Random Forest.

^c^GBM: gradient boosting method.

^d^AUC: area under the curve.

^e^XGBoost: extreme gradient boosting.

^f^LR: logistic regression.

^g^ANN: artificial neural network.

^h^GBDT: gradient boosting decision tree.

^i^ED: emergency department.

^j^LGBM: light gradient boosting method.

^k^RNN: recurrent neural network.

^l^DT: decision tree.

^m^WEKA: Waikato Environment for Knowledge Analysis.

^n^GLM: Generalized Linear Model.

^o^MLP: multilayers perceptron.

^p^LSTM: long short-term memory.

^q^NN: neural network.

^r^KNN: K-nearest neighbor.

### Model Explainability and Implementation

We then compared how model explainability was handled across studies. Model explainability refers to the degree of transparency and the level of detail a model can provide to offer additional information about its output, facilitating a better understanding of how the model operates [51]. We grouped the studies into 2 categories based on their best model’s transparency. In the first group, we included 18% (3/17) of the studies in which the best-performing model can be considered as a transparent model [51], including LR [23,24,34]. However, only 67% (2/3) of them provided a report on this model explanation in the form of LR coefficient values for each variable [23,34]. We grouped the remaining studies into an opaque model category where a post hoc analysis was needed to explain the model prediction mechanism [51]. In this group, all studies [4,11,22,26,28-33,35] used a model-specific method for explaining the prediction mechanism, except for 14% (2/14) of the studies [27,29] that used a model-agnostic method called the shapely additive explanation (SHAP) method [29]. Overall, 14% (2/14) of the studies in this group did not include any model explanation approach [21,25]. Although model-specific explanation methods, such as those used in DT-based models, gauge the impact of each feature on a model’s decision through specific metrics developed during training, the SHAP method takes a more comprehensive approach. SHAP conducts a deductive assessment by exploring all the potential combinations of features to determine how each one influences the final prediction.

None of the studies followed any specific reporting guidelines. Furthermore, despite promising performances in some studies, none were implemented in a real-world clinical setting for prospective evaluation. In each of the studies reviewed, various limitations were identified, encompassing both clinical and nonclinical factors. One of the common limitations in these studies was the issue of generalizing their findings to different health care settings and patient groups [22,25,26,29,33,35]. This difficulty often arose because they lacked important information such as medication histories [35], environmental factors [25,30], and social determinants of health [28], which are known to play significant roles in health outcomes. Data-related limitations were also prevalent, with some studies dealing with the drawbacks of structured EHR data [4,26,33,35], potential of data misreporting [32], and missing data that could affect the reliability of their models [29,35]. In addition, from a clinical perspective, certain studies faced limitations owing to the lack of standardized definitions for specific outcomes [11,22,23,27,28], emphasizing the importance of consistent criteria in health care research such as in asthma management. The model explanation and implementation information are summarized in Table 5. All data extraction results can be found in Multimedia Appendix 1. We have also depicted some of the important principal findings in Multimedia Appendix 2.

**Table 5:** Summary of model explainability and implementation.

Study, year	Best model transparency	Model explanation methods	Follow reporting guidelines	Clinical implementation	Study limitations
Inselman, et al [27], 2022	Opaque model	SHAP^a^	No	No	Missing relevant variables Limited data about different biologics Diverse primary uses of biologics Heterogeneity in patient characteristics
Hurst et al [25], 2022	Opaque model	No model explanation	No	No	Missing relevant variables Single-center study Location-dependent model performance Limited environmental data
Hogan et al [28], 2022	Opaque model	Estimated weights	No	No	Missing relevant variables Lack of longitudinal outcomes Use of *ICD-9*^b^ (older clinical coding) Hospital differentiation Absence of demographic data and social determinants
Zein et al [29], 2021	Opaque model	SHAP	No	No	Limited generalizability Reliance on diagnostic codes Limited clinical information Exclusion of anti-IL5^c^ therapy Cross-sectional nature Quality of clinical information Limited PFT^d^ and FeNO^e^ data Handling missing data
Sills et al [30], 2021	Opaque model	autoML method	No	No	Retrospective nature Patient selection criteria Limited clinical information Exclusion of home and environmental factors Timing of posttriage variables
Hozawa et al [31], 2021	Opaque model	Extracted risk factors	No	No	Age distribution discrepancy Limitations of claim data Prevalent user design Causality estimation
Lisspers et al [32], 2021	Opaque model	LGBM^f^ gain score	No	No	Data misreporting Applicability to other settings High false-positive rate Performance of shortlist model
Ananth et al [23], 2021	Transparent model	LR^g^ coefficients	No	No	Lack of formal asthma control assessment Limited longitudinal outcomes Lack of comorbidity information
Tong et al [33], 2021	Opaque model	XGBoost^h^ feature importance	No	No	Lack of relevant variables Nonuse of deep learning and unstructured data Expansion of data sources Generalizability across health care systems and diseases
Mehrish et al [24], 2021	Transparent model	No model explanation	No	No	Lack of relevant variables Limited method explanation
Xiang et al [4], 2020	Opaque model	Attention mechanism	No	No	Absence of complex interactions among clinical variables Limitations of structured EHR^i^ data Challenges in distinguishing symptoms and risk factors Opportunities for model enhancement
Cobian et al [34], 2020	Transparent model	LR coefficients	No	No	Limited samples
Luo et al [35], 2020	Opaque model	XGBoost feature importance	No	No	Lack of medication claim data Limitations of structured EHR data Opportunities for additional features Data completeness and generalizability
Roe et al [22], 2020	Opaque model	XGBoost feature importance	No	No	Intensive care setting exclusivity Exclusion of routine intensive care features Generalizability to outpatient settings
Luo et al [26], 2020	Opaque model	XGBoost feature importance	No	No	Potential unexplored features Nonuse of deep learning and unstructured data Limited generalizability assessment
Wu et al [21], 2018	Opaque model	No model explanation	No	No	Suboptimal neural network configuration Limited scope Clinical relevance and feature weighting
Patel et al [11], 2018	Opaque model	GBDT^j^ feature importance	No	No	Single institution data Pragmatic definition of the asthma population Lack of model validation Data limitations Lack of weather and CDC^k^ influenza data

^a^SHAP: shapely additive explanation.

^b^*ICD-9: International Classification of Diseases, Ninth Revision*.

^c^IL-5: interleukin 5.

^d^PFT: Pulmonary Function Tests.

^e^FeNO: Fractional Exhaled Nitric Oxide.

^f^LGBM: light gradient boosting method.

^g^LR: logistic regression.

^h^XGBoost: extreme gradient boosting.

^i^EHR: electronic health record.

^j^GBDT: gradient boosting decision tree.

^k^CDC: Centers for Disease Control and Prevention.

## Discussion

### Principal Findings

Our review indicates that this research field is still underdeveloped, given the limited body of evidence, heterogeneity of methods, lack of external validation, and suboptimally reported models. There was considerable heterogeneity in the specific definition of asthma outcome and the associated time horizon used by studies that sought to develop asthma attack risk prediction models. Class imbalance was also common across studies, and there was also considerable heterogeneity in how it was handled. Consequently, it was challenging to directly compare the studies.

The GBDT-based methods were the most reported best-performing method. DL methods such as long short-term memory (LSTM), a relatively more complex and advanced method, were also found in a few studies [4,21,34]. However, none of the studies compared the performance of the DL-based models with that of GBDT-based models. Moreover, none of the studies was prospectively evaluated or followed any reporting guidelines, and most studies (3/17, 18%) were not externally validated.

### Strengths and Limitations

The key strengths of our study include undertaking a systematic and transparent approach to ensure reproducibility. Overall, 2 independent reviewers followed a clear framework during the study selection and data extraction stage. Furthermore, the interpretation of the result was supported by a multidisciplinary team consisting of both technical and clinical experts.

A further strength is that most systematic reviews about the use of ML methods in asthma research have focused on either diagnosis or classifying asthma subtypes [52-56]. Although there have been 2 previous reviews about the use of ML in predicting asthma attacks [15,16], our review is the first to focus on several key considerations in an ML pipeline, from data preprocessing to model implementation for asthma attack predictions.

However, this review also has 3 key limitations. First, this scoping review provided broad coverage of various technical challenges, but it cannot ascertain how feasible and effective an ML-based intervention can be in supporting asthma management. Second, we were not able to directly compare studies owing to the heterogeneity across studies, and that prohibited us from identifying the best algorithm or approach for solving the technical challenges highlighted in this review. Finally, this review only focused on the technical challenges without taking into account additional, crucial, sociocultural and organizational barriers to the adoption of ML-based tools in health care [57-59].

### Interpretation in the Light of the Published Literature

The heterogeneity of outcome definitions found in this paper was also uncovered in previous non-ML asthma attack prognosis studies [16,60]. This heterogeneity includes both the indicators they used to define asthma attacks and the prediction time resolution. Recent systematic reviews also highlighted the wide range of outcome variations in ML-based prognostic models for ischemic stroke [61] and brain injury [62].

GBDT methods, especially XGBoost, have become a state-of-the-art method, especially for large and structured data in several domains [63-65]. Among the DL methods, LSTM has also shown potential in several previous studies [66,67]. LSTM is one of the most popular methods for analyzing complex time series data. Its capability to learn the sequence pattern makes it very powerful to build a prediction model by representing the data as a sequence of events. EHR data consist of a sequence of historical clinical events, which represent the trajectory of each patient’s condition over time. Incorporating the temporal features into the model, rather than just summarizing the events, can potentially boost the model’s performance.

Most of the studies (14/17, 82%) in this review incorporated some form of model explainability that aimed to provide an accessible explanation of how the prediction is derived by the model to instill trust in the users [68]. Previous studies in various domains showed that an ML model can output a biased prediction caused by latent characteristics within the data [69]. Model explainability is therefore crucial to provide model transparency and enhance fairness [70], especially in high-stake tasks such as those in health care [71].

Model validation and standard reporting are some of the important challenges that can influence adoption into routine practices [72]. An ML model should be internally, externally, and prospectively validated to assess its robustness in predicting new data [73]. In addition, a standard guideline needs to be followed in reporting an ML model development [74] such as the Transparent Reporting of a Multivariable Prediction Model for Individual Prognosis or Diagnosis [75] or the Developmental and Exploratory Clinical Investigations of Decision Support Systems Driven by Artificial Intelligence [76]. It will facilitate an improved and objective understanding and interpretation of model performance. However, our review found a lack of external validation and adherence to reporting guidelines among the selected studies. These points resonated with the findings in other reviews of different cases [77,78].

### Implications for Research, Policy, and Practice

This review highlighted several technical challenges that need to be addressed when developing asthma attack risk prediction algorithms. Further studies are required to develop a robust strategy for dealing with the class imbalance in asthma research. Class imbalance has been a common problem when working with EHR data [79,80]. However, there remains a notable gap in the literature regarding a systematic comparison of the effectiveness of existing methods, particularly in the context of asthma attack prediction. Several simple ML algorithms, such as linear regression, LR, and simple DTs, are easily interpretable [81]. In general, however, there is a trade-off between model interpretability and complexity, and most advanced methods are difficult to interpret, which then influences the users’ perception and understanding of the model [82]. We believe that the black box nature of the more complex methods, such as XGBoost and LSTM, is likely a technical barrier to implementing such models in a real-world clinical setting. Consequently, there is a need to continue exploring model explainability methods such as the attention mechanism approach recently developed for LSTM [83-85] that can augment complex “black box” algorithms.

There is a need for developing a global or at least a nationwide benchmark data set to facilitate external validation and to test the model’s generalizability [86]. Such validation is needed to ensure that the model will not only perform well under the data used in the model development but also can be reproduced to predict new data from different settings [87]. In addition, to maintain the transparency and reproducibility of the ML-based prediction model, adherence to a standard reporting guideline such as the Transparent Reporting of a Multivariable Prediction Model for Individual Prognosis or Diagnosis [75] should be encouraged. Both good reproducibility and clear reporting are key points to facilitate critical assessment of the model before its implementation into routine practices. This effort is pivotal in addressing ethical concerns associated with data-driven prediction tools and in guaranteeing the safety and impartiality of the prediction [88]. Ensuring the ethical aspects of integrating a data-driven model into routine clinical practice is becoming a great challenge. This task demands substantial resources and relies on a collaborative effort involving experts from various disciplines [89].

Finally, to ensure that the ML-based model meets the requirements of the practices, a clear use case must be articulated. We found that almost all studies follow a clear clinical guideline to define asthma attacks, but there is a wide range of prediction time horizons across the studies. These variations are the result of distinct needs and goals from different practices. It is impossible to make a one-size-fits-all model. Therefore, a clear and specific clinical use case should be defined as the basis for developing an ML-based model.

### Conclusions

ML model development for asthma attack prediction has been studied in recent years and includes the use of both traditional and DL methods. There is considerable heterogeneity in ML pipelines across existing studies that prohibits meaningful comparison. Our review indicates several key technical challenges that need to be tackled to make progress toward clinical implementation such as class imbalance problem, external validation, model explanation, and adherence to reporting guidelines for model reproducibility.

## Appendix

Multimedia Appendix 1 List of the search keywords and full data extraction result.

Multimedia Appendix 2 Key findings.

Multimedia Appendix 3 Preferred Reporting Items for Systematic Reviews and Meta-Analyses extension for Scoping Reviews (PRISMA-ScR) checklist.

## Abbreviations

DL: deep learning
DT: decision tree
EHR: electronic health record
GBDT: gradient boosting decision tree
LR: logistic regression
LSTM: long short-term memory
ML: machine learning
PRISMA: Preferred Reporting Items for Systematic Reviews and Meta-Analyses
SHAP: shapely additive explanation
XGBoost: extreme gradient boosting
