# Supplementary material for: Machine Learning–Based Asthma Attack Prediction Models From Routinely Collected Electronic Health Records: Systematic Scoping Review
Source: JMIR AI. 2023 Dec 7;2:e46717. doi: 10.2196/46717 (PMC11041490; doi:10.2196/46717)
Supplement: Multimedia Appendix 2 [file ai_v2i1e46717_app2.pdf]

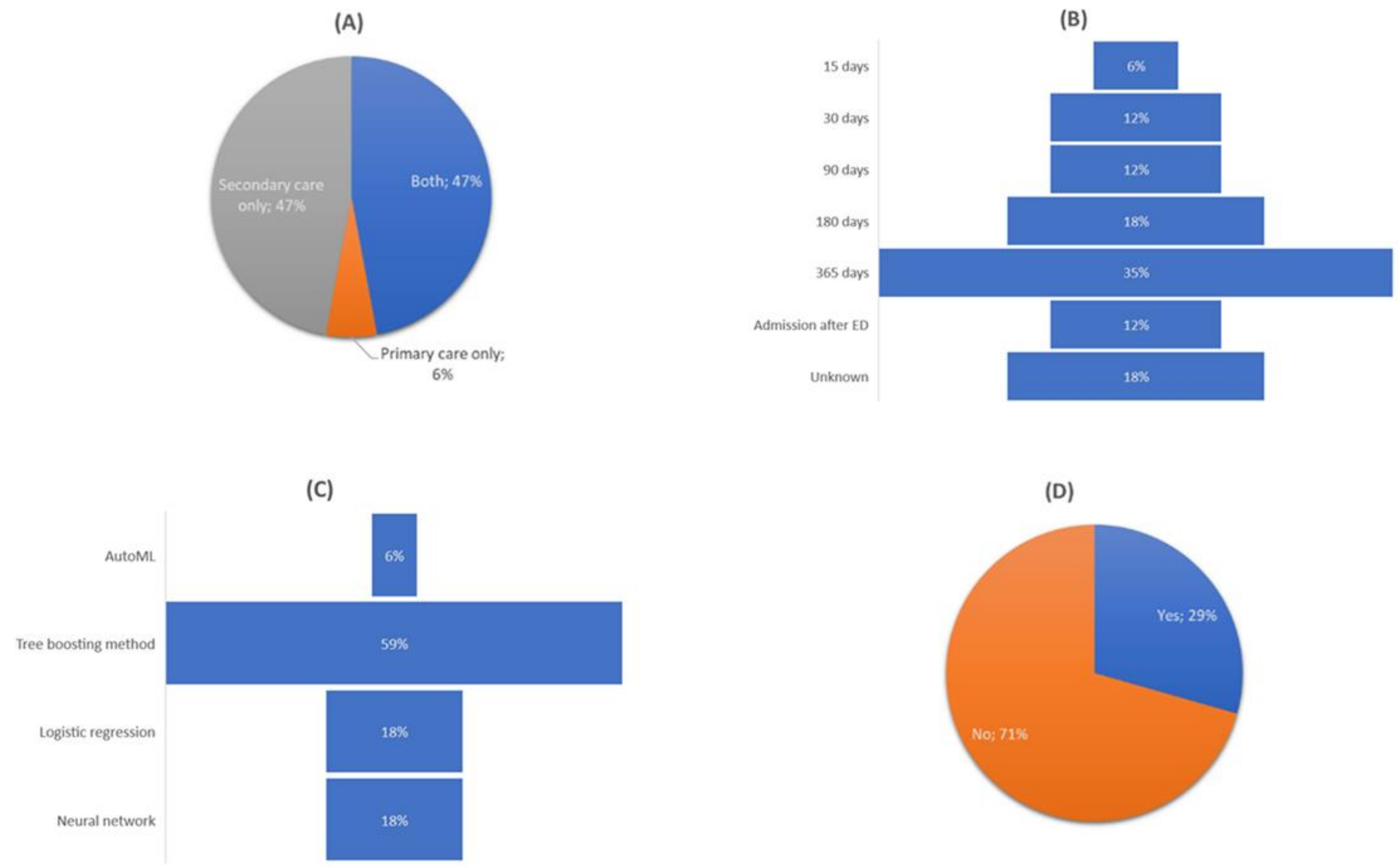

Figure 1. Distribution of the included studies in terms of (A) health care setting, (B) prediction time horizon, (C) best machine learning model, and (D) whether any method was reported to address class imbalance. ED: emergency department.
